# Myeloid sarcoma of the uterine cervix presenting as missed abortion

**DOI:** 10.1007/s00404-012-2454-8

**Published:** 2012-07-12

**Authors:** Harinder Gill, Florence Loong, Vivien Mak, Karen Chan, Wing-Yan Au, Yok-Lam Kwong

**Affiliations:** 1 Department of Medicine, Professorial Block, Queen Mary Hospital, Pokfulam Road, Hong Kong, China; 2 Department of Pathology, Queen Mary Hospital, Hong Kong, China; 3 Department of Medicine, Princess Margaret Hospital, Hong Kong, China; 4 Department of Obstetrics and Gynaecology, Queen Mary Hospital, Hong Kong, China

**Fig. 1:**
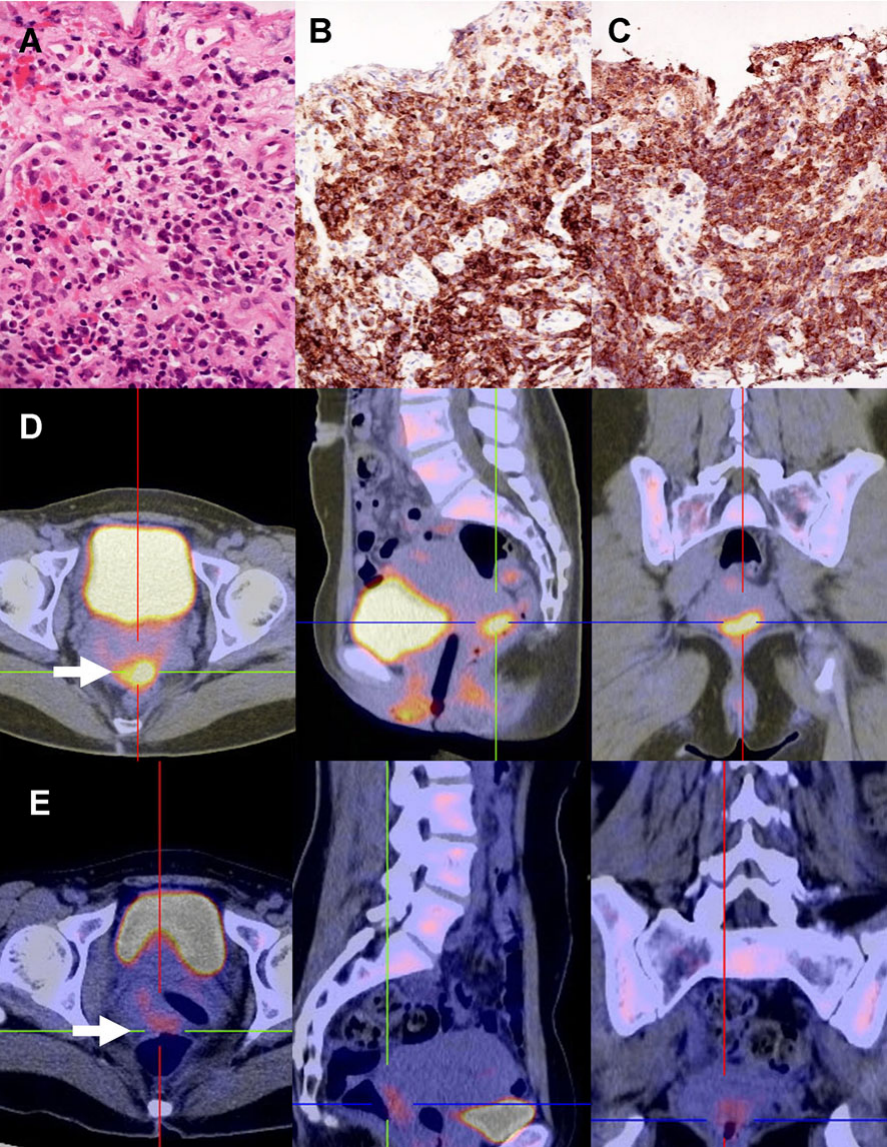
Myeloid sarcoma of the uterine cervix. **a** Biopsy showing medium-sized neoplastic cells intermixed with eosinophil precursors (H & E). **b** Neoplastic cells expressed CD45 (leucocyte common antigen) (immunoperoxidase). **c** Neoplastic cells expressed myeloperoxidase (immunoperoxidase). **d** Positron emission tomography, showing a hypermetabolic lesion (*arrow*) at the uterine cervix. **e** Post-treatment scan, showing diminution of the size and metabolic activity of the lesion

Dear Editor,

A 36-year-old woman (gravida 1, para 0) at 9 weeks of gestation presented with vaginal bleeding and a non-viable fetus. Medical abortion was performed. Histologic examination showed normal product of gestation. However, vaginal bleeding persisted. During dilatation and curettage, a 1 cm plaque-like lesion at the posterior cervical lip was found. The biopsy showed an atypical mononuclear cellular infiltrate intermixed with eosinophil precursors (Fig. 1a). These neoplastic cells were medium sized, with a high nuclear/cytoplasmic ratio and fine chromatin pattern, consistent morphologically with blasts. Immunohistochemical staining showed that the blast cells were positive for CD45 (leucocyte common antigen) (Fig. 1b) and the myeloid marker myeloperoxidase (Fig. 1c), but were negative for CD34 and CD117. The overall features were consistent with myeloid sarcoma. The blood count, bone marrow aspirate, and karyotype were normal. A positron emission tomography–computed tomography (PET–CT) showed a 3.3 × 1.7 cm lesion at the uterine cervix, with standard uptake value maximum (SUV_max_) of 7.4 (Fig. 1d). Another hypermetabolic focus (SUV_max_ 3.0) was noted at the left pelvic cavity, compatible with disease involvement. She was treated with a standard induction regimen for acute myeloid leukemia (AML) (cytarabine: 7 days; daunorubicin: 3 days). After 3 weeks, a reassessment PET–CT showed reduction of the cervical lesion to 2.5 × 1.3 cm with an SUV_max_ of 3.2 (Fig. 1e), consistent with partial response. She was treated with six further cycles of consolidation chemotherapy (etoposide: 5 days; daunorubicin: 2 days for two cycles; high dose cytarabine for four cycles). A reassessment PET–CT then showed complete response. The patient has remained in complete remission 16 months after diagnosis.

Myeloid sarcoma is rare, characterized by the myeloblasts presenting as tumor masses at extramedullary sites [1]. It may occur de novo, concurrently with AML or as blastic transformation of myelodysplastic syndrome or myeloproliferative neoplasm [2]. Frequent sites include: the skin, lymph nodes, mediastinum, gastrointestinal tract, bones, and testis [1, 2]. Myeloid sarcoma in the gynecological tract is very unusual, ovary and uterine cervix being involved in equal frequencies [3]. Around two-third of the cases had an antecedent myeloid neoplasms or concurrent AML [3–5]. Cervical myeloid sarcoma almost invariably presented as a mass lesion showing extensive tissue infiltration, manifesting as abdominal pain and vaginal bleeding [3–5].

Our case was special in several aspects. The disease was de novo. Interestingly, it presented as a missed abortion, an association not hitherto reported. Although examination showed a small plague, PET–CT revealed more extensive involvement, suggesting that the myeloid sarcoma might be causally related to abortion. PET–CT also showed involvement outside the uterus, so that it was instrumental in delineating the disease extent. As cervical carcinoma is far more common, and clinical presentation is similar, careful histopathologic evaluation, and awareness of the possibility of myeloid malignancies are required for the diagnosis of myeloid sarcoma.

The prognosis of cervical myeloid sarcoma appears poor, with survival in <20 % of the cases [3, 4]. The favorable outcome of our case might be related to early diagnosis, further underlining the importance of recognition of this pathology in the uterine cervix.
